# Infective endocarditis caused by *Arcanobacterium haemolyticum*: a case report

**DOI:** 10.1186/1476-0711-10-17

**Published:** 2011-05-12

**Authors:** Vanessa Wong, Tom Turmezei, Maria Cartmill, Shiu Soo

**Affiliations:** 1 Department of Microbiology, Queen's Medical Centre, Derby road, Nottingham, NG7 2UH, UK; 2 Department of Radiology, Queen's Medical Centre, Derby road, Nottingham, NG7 2UH, UK; 3 Department of Neurosurgery, Queen's Medical Centre, Derby road, Nottingham, NG7 2UH, UK

## Abstract

*Arcanobacterium haemolyticum *is an organism that commonly causes pharyngitis and wound infections. It does not usually cause systemic invasive disease. The organism presents a difficult diagnostic problem because the Clinical Microbiology laboratory has a propensity to view them as diphtheroid organisms of the *Corynebacterium *species, thus contaminants or normal flora. We describe a case of a 21-year-old female who had endocarditis with cerebral emboli due to *Arcanobacterium haemolyticum*. This rare condition is associated with significant mortality and to the best of our knowledge; this is the first successfully treated case of *A. haemolyticum *endocarditis complicated by embolic phenomenon.

## Introduction

*Arcanobacterium haemolyticum *is a facultative anaerobic gram-positive bacillus. Originally classified in the *Cornyebacterium *genus, it was re-classified in a new genus in 1982 [1]. It has been isolated from the skin and pharynx of healthy individuals and it is a well-recognised cause of pharyngitis, skin and soft-tissue infections [2]. Less commonly, the organism causes deep-seated infections including osteomyelitis [3], brain abscesses [4] and endocarditis [5-7].

*Arcanobacterium *means 'mysterious bacterium', which is an epithet quite befitting for an organism that is frequently overlooked by the Clinical Microbiology laboratory because it is deemed to be a contaminant or normal flora [8]. Recognition of the ability of this organism to cause disease is important in order to make a correct diagnosis and commence appropriate antibiotic therapy.

Here we describe the first case of successfully treated *A. haemolyticum *infective endocarditis complicated with cerebral emboli and review the features of this rarely pathogenic organism.

## Case report

A 21-year-old Caucasian female with known congenital heart disease presented with a five-day history of fever, lethargy and a swollen, painful left calf. Her past medical history included previous surgical repair for pulmonary atresia, quadricuspid aortic valve and a ventricular septal defect (VSD) as a child for which she took life-long warfarin anticoagulation therapy. She had also suffered recurrent miscarriages.

On examination she was febrile at 38.5°C, but hemodynamically stable. Significant positive findings were a swollen, tender left lower limb and an ejection flow murmur across the prosthetic aortic valve and pulmonary regurgitation through the pulmonary conduit, consistent with her heart condition. The remainder of the clinical examination was unremarkable.

Laboratory results showed: hemoglobin 9.3 g/dl; mean cell volume 0.32 fl; white cell count 8.6 × 10^9^/liter; neutrophil count 6.7 × 10^9^/liter; platelet count 268 × 10^9^/liter; C-reactive protein 35 mg/liter; INR 3.0. Her renal and liver function tests were normal. The admission chest radiograph was unremarkable. Ultrasound of her calf showed a hematoma, which was aspirated percutaneously under ultrasound guidance, the subsequent culture being negative.

**Figure 1:**
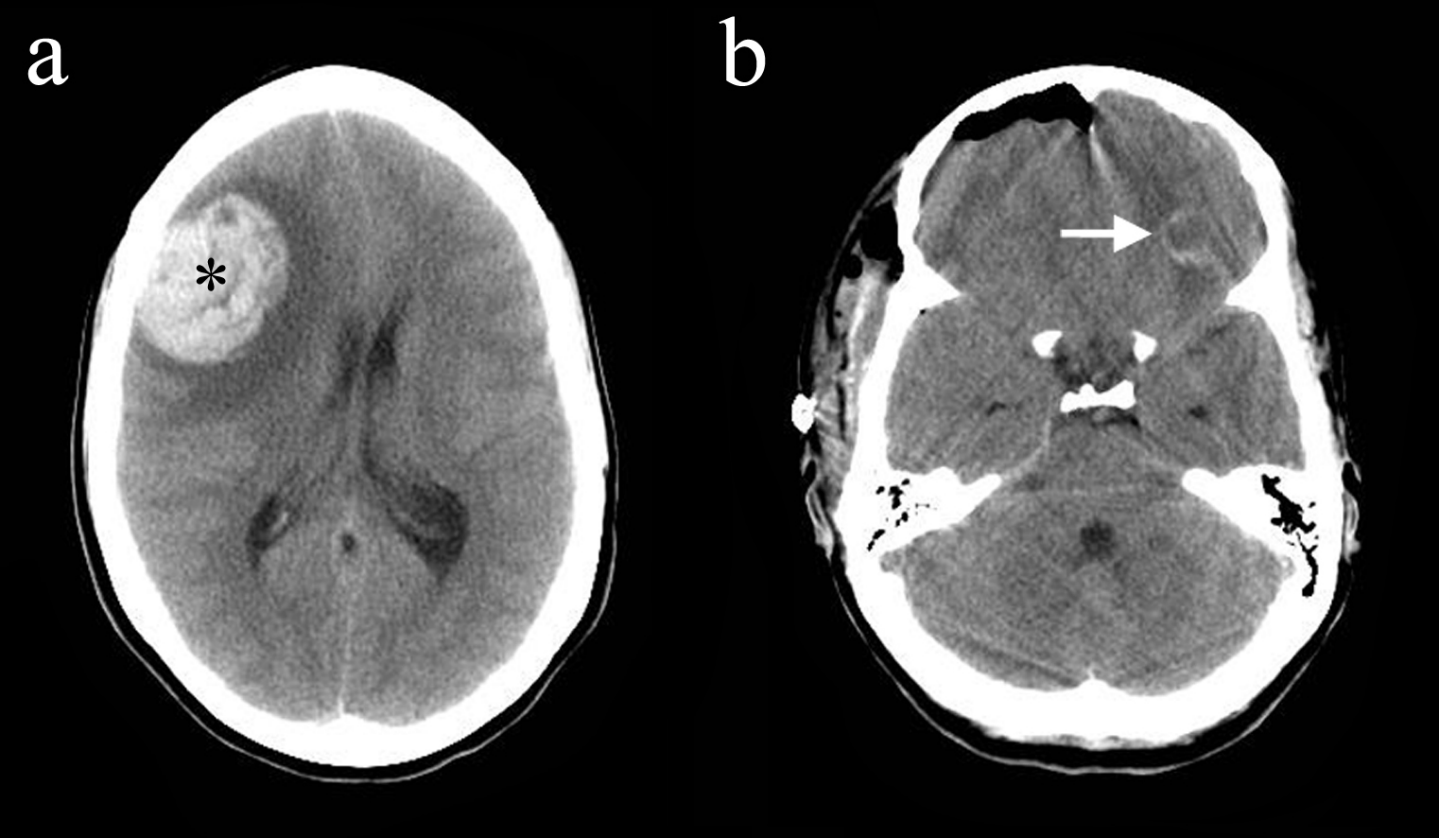
**a Axial image from the initial unenhanced CT brain study showing a large heterogeneous high density lesion in the right frontal brain parenchyma consistent with an acute hematoma (asterisk) with the surrounding low density of vasogenic edema causing midline shift to the left**. The presence of several smaller ring lesions (see **b**) combined with the clinical history led the reporting neuroradiologist to raise the possibility of intracranial mycotic aneurysms as well as cerebral abscesses. **b **Unenhanced axial CT brain image post-craniotomy and hematoma evacuation (note the pneumocephalus). This demonstrates one of the ring lesions in the inferior left frontal region (arrow) that was noted in the initial study. This showed rim enhancement with intravenous contrast administration (not shown), in keeping with the diagnosis of a cerebral abscess.

However, due to ongoing fevers the patient underwent a transthoracic echocardiogram (the patient was initially unable to tolerate the transesophageal approach) that raised the possibility of vegetations on the mitral valve and VSD patch. The patient then suffered a grand mal seizure requiring intubation and admission to the Intensive Care Unit. On examination a fixed and dilated right pupil was noted. Computerized tomography of the head showed a large right frontal parenchymal hematoma with several smaller frontal abscesses associated with mass effect and midline shift to the left (Figure 1a).

Neurosurgical review led to an emergency right frontal craniotomy with evacuation of the hematoma (Figure 1b), which was negative on microbiological culture. She was commenced on empirical intravenous ceftriaxone (2 g every 12 h), vancomycin (1 g every 12 h) and metronidazole (500 mg every 8 h) on advice from the Microbiology Department. Her post-operative progress was complicated by the need for further neurosurgery due to persistently raised intracranial pressure, resulting in a craniostomy.

Subsequently, 15 days into her admission one out of a total of 12 sets of blood culture specimens became positive after 22 hours of incubation on the BACTEC 9240 system (Becton Dickinson Microbiology Systems Ltd, USA). Pleomorphic gram-positive bacilli were seen in the blood gram film. The blood was inoculated onto several agar plates including: 5% horse blood agar (HBA; CM0271, Oxoid, UK); fastidious anaerobic agar (FAA; Oxoid, UK); Columbia blood agar with neomycin (FNEO; Oxoid, UK) aerobically and anaerobically for 2 and 5 days respectively. After 48 hours at 37°C both aerobic and anaerobic cultures yielded small translucent, non-pigmented colonies, surrounded by a small zone of beta-hemolysis on horse blood agar. The organism was noted to be catalase-negative and was identified as *A. haemolyticum *(99.9% probability) by biochemical testing with the API CORYNE identification strips (BioMérieux, France). This was later confirmed by the Health Protection Agency in London, United Kingdom, using 16S rRNA gene sequencing.

Antibiotic susceptibility testing by disk diffusion assay demonstrated that the organism was susceptible to penicillin, ceftriaxone, ciprofloxacin, gentamicin, rifampicin, and vancomycin. The minimal inhibitory concentration (MIC) of penicillin was 0.023 mg/L to the isolate (penicillin control MIC was 1.5 mg/L). There have been several reports of *A. haemolyticum *infective endocarditis in which there have been treatment failures with penicillin [5-7]. In view of this and the fact the patient was clinically improving, Microbiology advised continuing ceftriaxone with the addition of gentamicin (80 mg every 12 hours) to the treatment regime. Vancomycin and metronidazole were stopped at this point, having now received two weeks therapy of these. A course of 42 days of antibiotics was given in total, with all repeat blood cultures being negative.

On completion of the antibiotic course, the patient had some mild residual weakness and resting tremor of her right lower limb. Repeat transthoracic and transesophageal echocardiograms showed no evidence of endocarditic phenomena. At six months follow-up the patient reported no new symptoms and there was no clinical evidence of relapse of infective endocarditis. Subsequently the patient underwent a successful insertion of intracranial titanium plates and titanium cranioplasty nine months later.

## Discussion

*Arcanobacterium haemolyticum *was first isolated from pharyngitis and skin infections in American soldiers and native islanders in the South Pacific in 1946 [9]. The organism is often overlooked as part of the normal oral flora, however it has been found in symptomatic individuals either as a sole pathogen or a component of polymicrobial infection [10,11]. No risk factors for infection have yet been identified [4,12], although two distinct patient subsets are recognized: healthy young adults presenting with upper respiratory tract infections and older, often immunocompromised, patients presenting with skin and soft tissue infections [11].

Although *A. haemolyticum *can cause infective endocarditis, to the best of our knowledge only three cases have been reported in the medical literature: the first case was an 87-year-old gentleman who died after developing an infection on a bicuspid aortic valve [7]; the second case was a 50-year-old intravenous drug user (IVDU) who developed mitral valve infective endocarditis and died as a result of cerebral emboli [6] and the third case was a 33-year-old IVDU with HIV-1 infection who survived after developing infective endocarditis of his tricuspid valve without any complication [5]. Our case report describes a young woman with congenital heart disease that survived despite developing neurological complications requiring surgery.

After 48 hours incubation colonies display beta-hemolysis that is best observed on 5% human blood agar [13]. The growth can be optimized by the presence of 5-8% carbon dioxide, blood or serum-enriched medium and incubation at 37°C [14]. There are two distinct biotypes of *A. haemolyticum*: smooth or rough colonies on solid growth medium. Smooth-type colonies appear even, beta-hemolytic, beta-glucuronidase negative and ferment both sucrose and trehalose. Rough-type colonies appear uneven, non-hemolytic, beta-glucuronidase positive, and do not ferment sucrose or trehalose. The majority of strains are of the smooth-type. Clinically there is a difference between the biotypes with the smooth-type predominately causing wound infections, while the rough-type is isolated almost exclusively from respiratory specimens [15].

There are no established guidelines for the treatment of this infection, although it has been reported to be sensitive to penicillin, cephalexin, erythromycin, and clindamycin, yet resistant to sulfamethoxazole-trimethoprim [16]. Cephalosporins have been found to be reasonable first line agents for deep-seated infections because they are bactericidal. They have good tissue penetration in systemic infections and low rates of resistance have been reported [14]. In addition, the organism has also demonstrated high susceptibility to gentamicin [17]. There have been reports of treatment failure with penicillin, with penicillin tolerance being described on *in vitro *testing. In infective endocarditis *A. haemolyticum *has also demonstrated both penicillin and ampicillin tolerance, resulting in no clinical improvement on empirical therapy of ampicillin plus gentamicin [5]. Therefore it is prudent to be aware of tolerance to penicillins when deciding on an antibiotic regime with this organism.

## Conclusions

Given the serious morbidity associated with disseminated *A. haemolyticum *infection and the difficulties that face a Clinical Microbiology laboratory in recognizing it as a human pathogen, it is important to be aware of its potential role in causing bloodstream infections, particularly in patients with predisposing factors.

## Competing interests

The authors declare that they have no competing interests.

## Authors' contributions

VW and TT participated in the concept and design of the manuscript, acquisition of data, and drafting of the manuscript. All authors read and approved the final manuscript.

## Consent

Written informed consent was obtained from the patient for publication of this case report and any accompanying images. A copy of the written consent is available for review by the Editor-in-Chief of this journal.
